# Detection of asymptomatic recurrence following curative surgery improves survival in patients with gastric cancer: A systematic review and meta-analysis

**DOI:** 10.3389/fonc.2022.1011683

**Published:** 2022-10-26

**Authors:** Hua-Yang Pang, Meng-Hua Yan, Li-Hui Chen, Xiu-Feng Chen, Zhi-Xiong Chen, Shou-Ru Zhang, Hao Sun

**Affiliations:** Department of Gastroenterology, Cancer Hospital, Chongqing University, Chongqing, China

**Keywords:** gastri cancer, intensive follow-up, recurrence, survival outcomes, meta-analysis

## Abstract

**Background:**

To date, there is no evidence that intensive follow-up provides survival benefit in gastric cancer patients undergoing curative gastrectomy. The aim of this study is to investigate the efficacy of detection of asymptomatic recurrence using intensive surveillance strategy in long-term survival after curative gastric cancer surgery.

**Methods:**

A systematic review of electronic databases including PubMed, Embase, Web of Science, the Cochrane Library and China National Knowledge Infrastructure, Clinical Trials Registry and Google Scholar was performed up to April 2022. The primary outcomes were survival outcomes: overall survival, recurrence-free survival and post-recurrence survival. The secondary endpoints were clinicopathological features, recurrence patterns and treatment after recurrence. The registration number of this protocol is PROSPERO CRD42022327370.

**Results:**

A total of 11 studies including 1898 participants were included. In the pooled analysis, the detection of asymptomatic recurrence was significantly associated with an improved overall survival compared to patients showing symptoms of recurrence (HR=0.67; 95%CI: 0.57-0.79; P<0.001), which was primarily driven by the prolongation of post-recurrence survival (HR=0.51; 95%CI: 0.42-0.61; P<0.001), since there was no significant difference observed in recurrence-free survival (HR=1.12; 95%CI: 0.81-1.55; P=0.48) between the two groups. Meanwhile, male sex and advanced T stage were more frequently observed in the symptomatic recurrence group. Furthermore, patients in the symptomatic recurrence group had a higher proportion of peritoneal relapse but lower proportion of distant lymph node metastasis. Additionally, patients in the symptomatic recurrence group were less likely to receive surgery treatment and post-recurrence chemotherapy.

**Conclusion:**

The detection of asymptomatic recurrence using intensive follow-up was associated with an appreciable improvement in overall survival. However, more robust data from high-quality studies are still required to verify this issue.

**Systematic review registration:**

https://www.crd.york.ac.uk/prospero/display_record.php?RecordID=327370, identifier CRD42022327370.

## Background

Gastric cancer (GC) is still one of the most common causes of cancer-related deaths worldwide, especially in East Asia (1, 2). Once the GC is detected, surgery with curative intent is the treatment choice for nonmetastatic disease (3, 4). However, despite curative resection was performed, 70%-80% GC recurrences occur within the first 2 years and about 90% of recurrences occur by 5 years, which makes the prognosis of GC patients poor (5, 6).

Currently, international guidelines from the National Cancer Comprehensive Network (NCCN) (7), the Japanese Gastric Cancer Association (JGCA) (8) and the European Society for Medical Oncology (ESMO) (8) consistently recommend postoperative intensive follow-up strategies for GC survivors, despite no consensus on the frequency and regimen of surveillance. Intensive follow-up is beneficial to manage treatment-related complications and collect data (9, 10). Most importantly, intensive follow-up is considered to enable early detection of recurrence disease, therefore instigating further treatment without delay and improving survival outcomes of these patients (11). Consequently, intensive follow-up is actively recommended by clinicians, aiming to detect recurrence as soon as possible, preferably before tumor-related symptoms develop (6).

In colorectal cancer, several meta-analyses have demonstrated improved survival in patients with asymptomatic recurrence (AR) using intensive follow-up strategies compared to these who present later with symptomatic recurrence (SR) (12, 13). The same is true for lung cancer (14) and pancreatic cancer (15). Nevertheless, in gastric cancer, the literature regarding the benefits of intensive follow-up on survival is scarce and extremely inconsistent. Therefore, the JGCA guidelines (16) and the ESMO guidelines (8) clearly state that there is no evidence that intensive follow-up provides survival benefit to GC patients undergoing curative resection. Recently, a newly published meta-analysis based on five studies also failed to demonstrate the benefit of planned surveillance strategies in improving detection of recurrence and post-recurrence survival (PRS) in GC patients (17).

Herein, we performed a systematic review and meta-analysis based on previously published studies to investigate whether the detection of AR using intensive follow-up could improve the survival outcomes, especially overall survival (OS), in GC patients who underwent curative resection.

## Methods

The present study was conducted in line with the requirements from Preferred Reporting Items for Systematic Reviews and Meta-Analyses (PRISMA) guidelines to identify studies that assess the association of the detection of AR and survival outcomes in GC patients who underwent curative resection. The meta-analysis was registered in PROSPERO (CRD42022327370).

### Search strategy

Relevant studies from electronic datasets including PubMed, Embase, Web of Science, and the Cochrane Library were systematically examined up to April 1, 2022. In addition, with the same cutoff date, the Clinical Trials Registry and Google Scholar were used to search unpublished trials and grey literature, respectively. Language restrictions were not applied during the search process. The following combination of key words were used to identify potential studies: [“asymptomatic recurrence” OR “symptomatic recurrence” OR “intensive surveillance” OR “intensive follow-up”] AND [“gastric cancer” OR “gastric carcinoma” OR “gastric neoplasm” OR “stomach cancer” OR “stomach carcinoma” OR “stomach neoplasm”]. Additionally, the references of the included studies were searched for additional reports. The search was performed by two investigators independently (HY-P and MH-Y).

### Inclusion and exclusion criteria

The inclusion criteria were determined according to the PICOS approach as follows. P: Patients were pathologically diagnosed as gastric cancer and underwent curative resection; I: AR, which was defined as recurrence discovered by a routine laboratory, imaging, or endoscopic test that was not prompted by any clinical concerns; C: SR, which was defined as the presence of patient-reported symptoms triggering further investigations; O: Time-to-event survival outcomes; S: Comparative studies including randomized controlled trials, cohort and case-controlled studies.

The exclusion criteria were studies 1) reported as case reports, reviews, letters and abstracts; 2) with overlapping data; 3) whose data were not adequate for meta-analysis or unavailable from the authors.

### Data extraction and quality assessment

Two independent reviewers (HY-P and MH-Y) conducted the data extraction and cross-checked all the results. The extracted data included first author, year of publication, study interval, country, study design and sample size, clinicopathological features like age, sex, tumor location, histological differentiation, Lauren type, tumor stage, adjuvant chemotherapy, recurrence patterns, treatment after recurrence, follow-up time, and survival outcome measures.

The Newcastle Ottawa Scale (NOS) (18) was used to assess the quality of included observational studies and a study with NOS score >6 is regarded as of high quality.

### Outcomes

In the present study, the primary outcomes were to investigate the impact of detection of AR on survival outcomes, including OS (from the date of surgery to the date of any cause of death), PRS (from the date of recurrence to the date of any cause of death) and recurrence-free survival (RFS: from the date of surgery to the date of recurrence). Secondary outcomes were aimed to compare the clinicopathological features, recurrence patterns and treatment after recurrence between the two groups.

Of note, since disease free survival (DFS: from the date of surgery to the date of recurrence or any cause of death) and RFS share the similar endpoints, they were analyzed together as one outcome, RFS (19, 20).

### Statistical analysis

The hazard ratio (HR) and odds ratio (OR) with their 95% confidence intervals (CI) were used as the effect size for survival outcomes and dichotomous variables, respectively. For studies that HR with 95%CI was not reported, we then calculated them from the necessary data following the methods reported by Tierney et al. (21) Heterogeneity among studies was assessed using I (2) statistic. All pooled analyses were performed assuming the random-effects model, which accounts for variance across included studies. Subgroup analysis was performed to explore the source of heterogeneity. A leave-one-out sensitivity analysis was conducted to evaluate the robustness and credibility of the results. Publication bias was evaluated using Begg’s funnel plot. A P value <0.05 was considered statistically significant. All of these statistical analyses were performed by Review Manager Software, version 5.3 (Cochrane, London, UK) and Stata, version 12.0 (Statacorp, College Station, TX).

## Results

### Study characteristics

**Figure 1:**
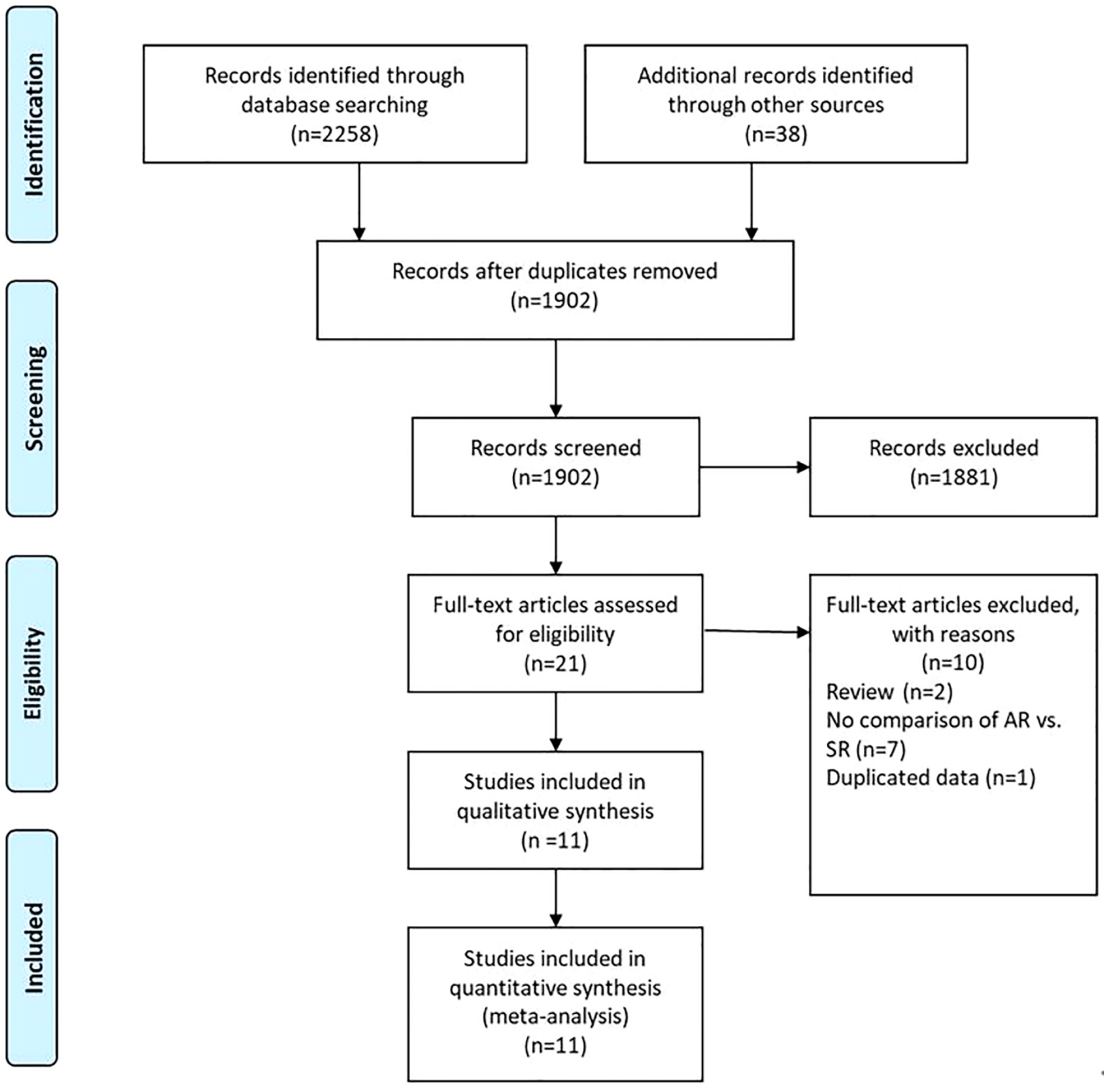
The PRISMA Flowchart of study selection.

A flow chart of the selection process was shown in **Figure 1**. The search strategy yielded 2296 potential studies. After title, abstract assessment and full text assessment, 11 retrospective cohort studies (22–32) were finally included in the present study. The characteristics of the included studies were shown in **Table 1**. A total of 1898 GC patients from 8 countries who underwent curative (R0) resection were included in this study. These studies were published from 2003 to 2022 with a sample size ranging from 47 to 382. Among these studies, the median follow-up time ranged from 22.5 to 169.8 months. With respect to the survival outcomes, the OS was reported in all of these included studies, while RFS/DFS and PRS were reported in 8 and 8 studies, respectively. The surveillance protocols of most studies were routinely based on the combination of history, physical examination, blood tests and imaging-based evidence mainly from CT. However, Moorcraft et al. (29) did not routinely perform imaging-based examination during the follow-up unless the recurrence was clinically suspected. The details of quality assessment of the included studies were shown in the **Supplementary File** (**Table S1**), and the scores of these studies ranged from 6 to 7 after careful assessment with NOS.

**Table 1:** Basic information of included studies.

Reference	Country	Study design	Study interval	Sample size(AR / SR)	Age, years(Median or mean)	Sex(Male/Female)	Tumor stage	Survival analysis	Median follow-up (months)	Surveillance protocol	NOS score
Bennett,2005 (22)	USA	Retro; S	1985-2000	382(99/283)	NA	267/115	I-IV	RFS*; PRS^#^; OS*	NA	Routinely imaging based	7
Bilici,2013 (23)	Turkey	Retro; S	2000-2010	173(100/73)	NA	118/55	I-III	OS*; DFS*	22.5	Routinely imaging based	6
Diniz,2022 (24)	Brazil	Retro; S	1999-2018	166(109/166)	NA	95/71	I-III	PRS *; OS*; DFS*	80.1	Routinely imaging based	7
Fujiya,2016 (25)	Japan	Retro; S	2002-2014	218(117/101)	69.0/66.0	150/68	II-IV	RFS*; PRS *; OS*	NA	Routinely imaging based	7
Kim,2010 (26)	Korea	Retro; S	2000-2004	110(55/55)	55.4/56.2	68/42	I-IV	RFS*; PRS ^#^; OS*	NA	Routinely imaging based	6
Kodera,2003 (27)	Japan	Retro; S	1985-1996	197(88/109)	62.0/60.0	133/64	I-IV	PRS *; OS*; DFS*	NA	Routinely imaging based	7
Mikami,2007 (28)	Japan	Retro; S	1995-2002	62(19/43)	NA	43/19	I-IV	PRS^#^	NA	Routinely imaging based	6
Moorcraft,2016 (29)	UK	Retro; S	2001-2010	47(13/34)	NA	NA	I-IV	OS^#^; DFS^#^	61.7	Imaging based on clinical suspicion	6
Park,2021 (30)	Korea	Retro; S	2002-2017	305(161/144)	57.8/59.1	207/98	I-III	PRS ^#^; OS^#^	169.8	Routinely imaging based	7
Villarreal-Garza,2011 (31)	Mexico	Retro; S	1980-2006	75(14/61)	54.2/54.2	43/32	I-III	RFS*; PRS *; OS*	43.0	Routinely imaging based	7
Zhao,2011 (32)	China	Retro; S	2000-2005	163(91/72)	NA	102/61	II-IV	OS^#^	NA	Routinely imaging based	7

AR, asymptomatic recurrence; SR, symptomatic recurrence; Retro, retrospective; S, single center; NOS, Newcastle Ottawa Scale; NA, not available; RFS, recurrence-free survival; PRS, post-recurrence survival; OS, overall survival; DFS, disease-free survival.

*: data extracted from survival curve; #, data derived from reported result.

### Overall survival

**Figure 2:**
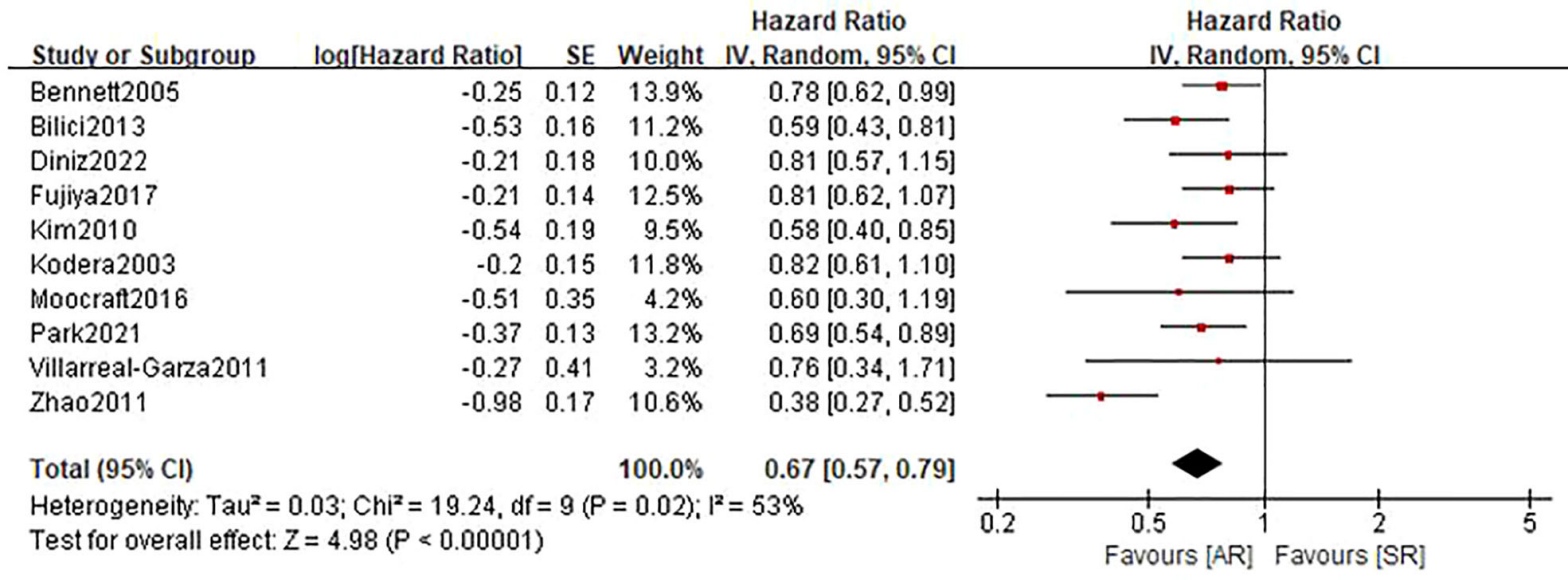
Forest plot assessing overall survival between the asymptomatic and symptomatic recurrence group.

Ten studies (22–27, 29–32) involving 1836 patients (795 in the AR group and 1041 in the SR group) reported the OS outcome. Briefly, five studies (22, 23, 26, 30, 32) showed patients in the AR group had a superior OS than patients in the SR group, but another five studies (24, 25, 27, 29, 31) showed no differences between the two groups. After adding these results, the pooled HR was 0.67 (95%CI: 0.57-0.79; P<0.001), which indicated that the detection of AR could improve the OS in GC patients undergoing curative resection (**Figure 2** and **Table 2**), although there was a moderate heterogeneity (I^2 ^= 53%; P=0.02). Subgroup analyses based on country, sample size, TNM stage, survival analysis, NOS score were performed and shown in **Table 3** and **Figure S1**. Despite heterogeneity in some subsets, the pooled results of almost all subgroup analyses revealed that patients in the AR group had a significantly better OS when compared with these in the SR group. Additionally, sensitivity analysis showed that the pooled outcome did not substantially change (**Figure S3A**).

**Table 2:** Baseline features and outcomes of AR patients vs. SR patients.

	Studies, n	Patients, n	OR (95%CI)	P value	I^2^ (%)
**Clinicopathological features**					
Sex (Male)	8	1407	1.45(0.15-0.81)	0.001	0
Tumor Location (Lower third)	5	721	1.02(0.74-1.39)	0.92	0
Histology (Poor differentiation)	3	466	0.73(0.31-1.72)	0.47	76
Lauren type (Diffuse type)	2	241	0.60(0.18-1.93)	0.39	66
T stage (III/IV)	6	939	0.67(0.49-0.92)	0.01	0
N stage (N+)	7	1102	1.09(0.76-1.56)	0.63	0
Adjuvant chemotherapy (Yes)	5	854	1.21(0.69-2.14)	0.51	74
**Recurrence patterns**					
Locoregional recurrence	8	1306	1.16(0.73-1.82)	0.53	36
Peritoneal recurrence	7	1109	0.38(0.19-0.77)	0.007	81
Hematogenous recurrence	6	926	1.52(0.71-3.30)	0.28	85
Distant lymph node metastasis	5	972	1.78(1.03-3.08)	0.04	52
**Treatment after recurrence**					
Tumor resection	6	1071	2.02(1.11-3.68)	0.02	39
Chemotherapy	6	1071	1.49(0.93-2.41)	0.10	59
Best support care	2	384	0.45(0.27-0.73)	0.001	0
No treatment	3	588	0.35(0.21-0.58)	<0.001	0
**Survival outcomes**					
Overall survival	10	1836	0.67(0.57-0.79)	<0.0001	53
Recurrence-free survival	8	1368	1.12(0.81-1.55)	0.48	83
Post-recurrence survival	8	1515	0.51(0.42-0.61)	<0.0001	57

AR, asymptomatic recurrence; SR, symptomatic recurrence.

**Table 3:** Subgroup analysis for overall survival and recurrence-free survival of AR patients vs. SR patients.

		Studies, n	Patients, n	HR (95%CI)	P value	I^2^ (%)
**Overall survival**						
	Total	10	1836	0.67(0.57-0.79)	<0.0001	53
Country	Eastern	5	993	0.64(0.49-0.84)	0.001	75
	Non-eastern	5	843	0.72(0.62-0.84)	<0.0001	0
Sample size	>200	3	905	0.76(0.65-0.88)	0.0002	0
	≤200	7	931	0.62(0.49-0.79)	<0.0001	60
TNM stage	I-III	4	719	0.69(0.58-0.81)	<0.0001	0
	I-IV	4	736	0.74(0.63-0.87)	0.0002	0
	II-IV	2	381	0.55(0.26-1.18)	0.13	92
Survival analysis	Univariate	7	1321	0.74(0.66-0.83)	<0.0001	0
	Multivariate	3	515	0.53(0.34-0.84)	0.006	76
NOS score	6	3	438	0.69(0.55-0.87)	0.002	19
	7	7	1398	0.67(0.54-0.82)	0.0001	64
**Recurrence-free survival**						
	Total	8	1368	1.12(0.81-1.55)	0.48	83
Country	Eastern	3	525	1.23(0.72-2.08)	0.45	89
	Non-eastern	5	843	1.06(0.65-1.74)	0.81	83
Sample size	>200	2	600	1.58(1.24-2.02)	0.0002	7
	≤200	6	768	1.00(0.69-1.44)	1.0	80
TNM stage	I-III	3	414	0.90(0.41-2.00)	0.80	90
	I-IV	4	736	1.13(0.81-1.58)	0.46	61
	II-IV	1	218	1.72(1.30-2.26)	0.0001	–
Survival analysis	Univariate	7	1321	1.09(0.77-1.54)	0.62	85
	Multivariate	1	47	1.48(0.76-2.88)	0.25	–
NOS score	6	3	438	1.07(0.44-2.61)	0.89	94
	7	5	930	1.15(0.87-1.52)	0.33	62

AR, asymptomatic recurrence; SR, symptomatic recurrence.

### Recurrence free survival

**Figure 3:**
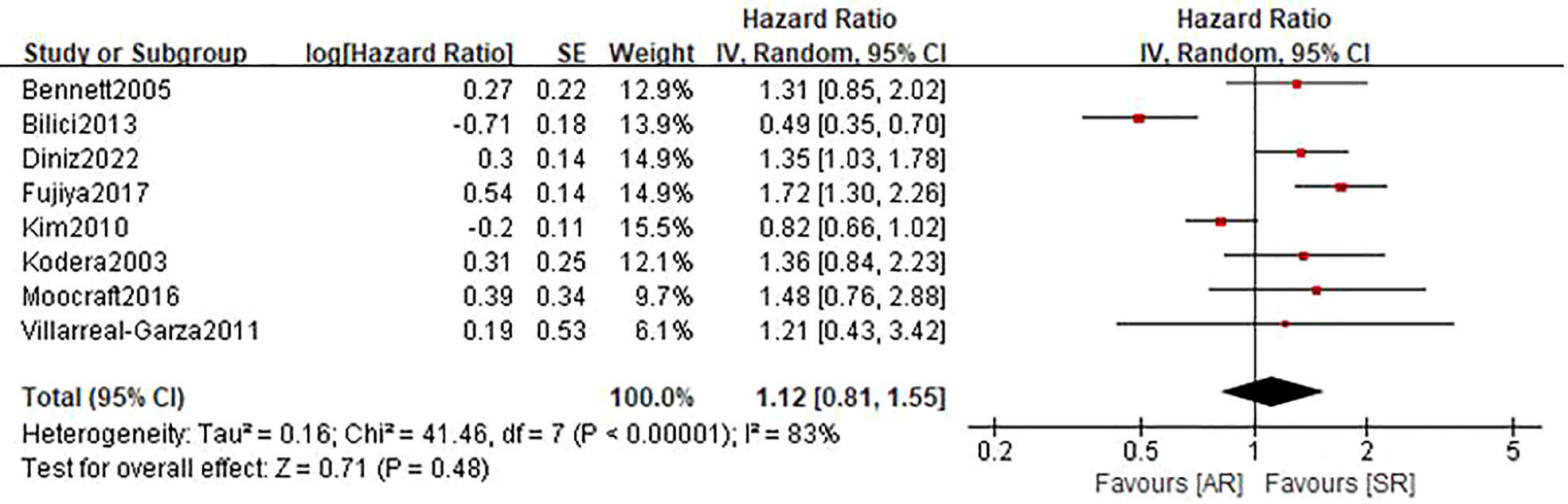
Forest plot assessing recurrence-free survival between the asymptomatic and symptomatic recurrence group.

A total of 8 studies (22–27, 29, 31) involving 1368 patients (543 in the AR group and 825 in the SR group) reported on RFS. Among these studies, one study (23) reported a favorable RFS in the AR group, 2 studies (24, 25) showed a better RFS in SR group, and the remaining 5 studies (22, 26, 27, 29, 31) did not show differences in this outcome. The pooled HR was 1.12 (95%CI: 0.81-1.55; P=0.48; I^2 ^= 83%), which suggested that there was no significant difference in the time to recurrence between the two groups (**Figure 3** and **Table 2**). Further subgroup analyses based on the above-mentioned parameters indicated that there were no significant differences in terms of RFS in most subgroups, except for the subgroup which had a sample size of more than 200 patients and the subgroup which included patients with stage II-IV disease, showed a favorable RFS in the SR group (**Table 3** and **Figure S2**). Sensitivity analysis indicated that the pooled outcome did not substantially change (**Figure S3B**).

### Post-recurrence survival

**Figure 4:**
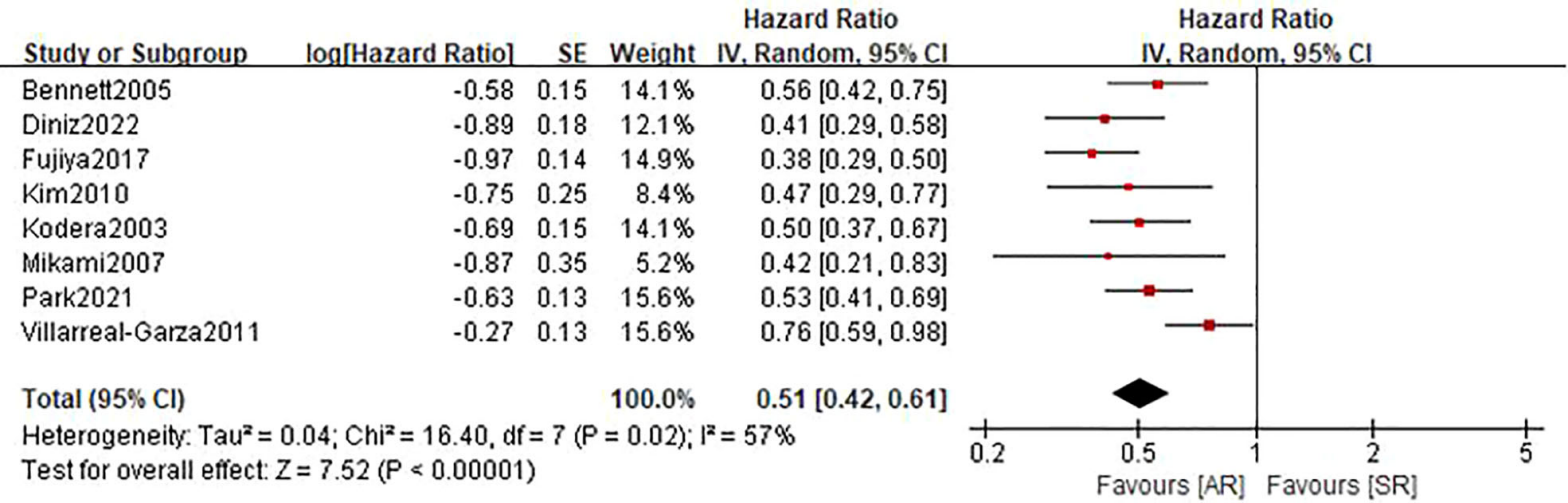
Forest plot assessing post-recurrence survival between the asymptomatic and symptomatic recurrence group.

Eight studies (22, 24–27, 30, 31) involving 1515 patients (610 in the AR group and 905 in the SR group) reported this outcome. The pooled HR was 0.51 (95%CI: 0.42-0.61; P<0.001), which suggested that the detection of AR was associated with an improved PRS (**Figure 4**). Even though there was a degree of heterogeneity (I^2 ^= 57%), all of these studies consistently concluded that the detection of AR helps improve PRS.

### Clinicopathological features

As shown in **Table 2** and **Figure S4**, pooled results showed that asymptomatic recurrence was significantly associated with a higher proportion of male (OR=1.45, 95%CI: 1.15-1.81, P=0.001; I^2 ^= 0%), and less commonly presented as T3/4 tumors (OR=0.67, 95%CI: 0.49-0.92, P=0.01; I^2 ^= 0%). No association was found between symptomatic recurrence with tumor location, histological differentiation, Lauren type, nodal metastasis and adjuvant chemotherapy. Pooled analysis for age was not feasible due to the lack of available data.

### Recurrence patterns

**Figure 5:**
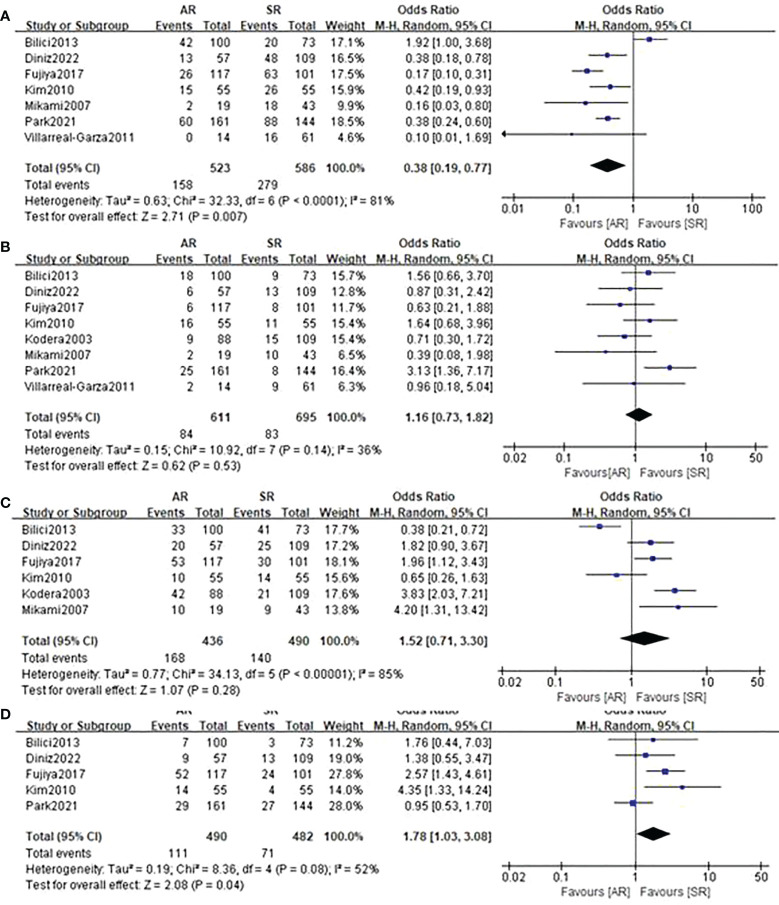
Forest plot assessing recurrence patterns including **(A)** peritoneal recurrence, **(B)** locoregional recurrence, **(C)** hematogenous recurrence, **(D)** distant lymph node metastasis between the asymptomatic and symptomatic recurrence group.

**Figure 5** presents results of specific recurrence patterns between the AR and SR groups. Compared with the SR group, the pooled analyses identified that patients in the AR group were more likely to occur distant lymph node metastasis (OR=1.78, 95%CI: 1.03-3.08, P=0.04; I^2 ^= 52%) but less likely to develop peritoneal relapse (OR=0.38, 95%CI: 0.19-0.77, P=0.007; I^2 ^= 81%). With respect to locoregional recurrence and hematogenous recurrence, there were no obvious differences observed between the two groups.

### Treatment characteristics

**Figure 6:**
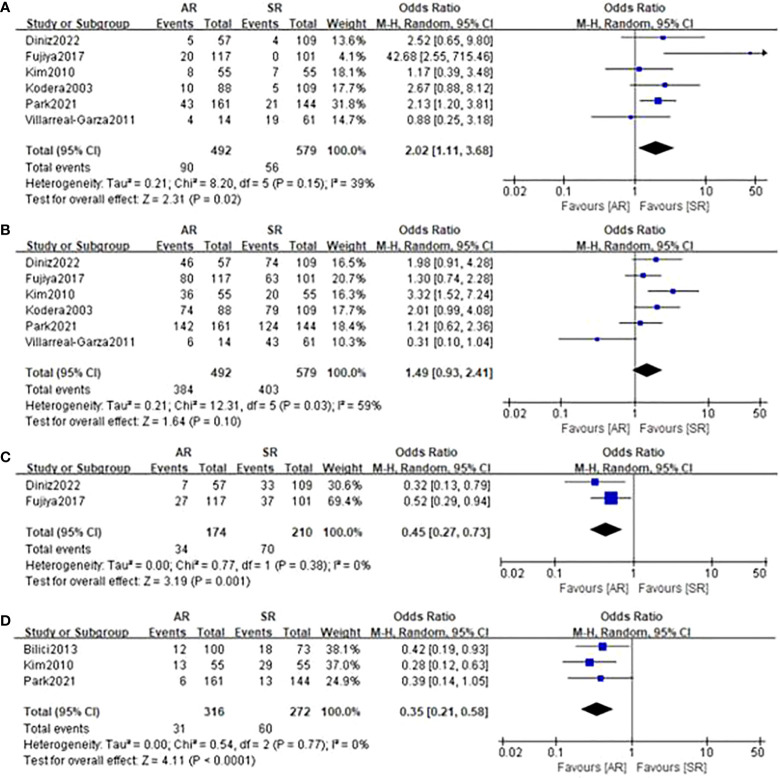
Forest plot assessing treatment after recurrence including **(A)** tumor resection, **(B)** chemotherapy, **(C)** basic support care, **(D)** no treatment between the asymptomatic and symptomatic recurrence group.

In terms of treatment after recurrence (**Figure 6**), the incorporated analyses revealed that patients in the AR group had a higher rate of surgery for recurrent lesions (OR=2.02, 95%CI: 1.11-3.68, P=0.02; I^2 ^= 39%) and a trend towards a higher rate of chemotherapy treatment (OR=1.49, 95%CI: 0.93-2.41, P=0.10; I^2 ^= 59%). On the contrary, the detection of AR could significantly decrease the proportion of patients receiving basic support care (OR=0.45, 95%CI: 0.27-0.73, P=0.001; I^2 ^= 0%) and no treatment (OR=0.35, 95%CI: 0.21-0.58, P<0.001; I^2 ^= 0%).

### Publication bias

The Begg’s funnel plot was performed to assess the potential publication bias of the primary outcomes. As shown in **Figure S5**, the funnel plots of OS, RFS and PRS were virtually symmetric, and the P values of Begg’s test were 0.371, 0.803 and 0.258, respectively, indicating that these pooled outcomes had a low risk of publication bias.

## Discussion

This systematic review and meta-analysis demonstrated that asymptomatic GC patients undergoing intensive follow-up after curative gastrectomy had a significantly longer OS than patients showing symptoms of recurrence. The survival benefit was primarily driven by the prolongation of PRS, since there was no significant difference observed in RFS between the two groups. Meanwhile, male sex and advanced T stage were more frequently observed in the SR group than in the AR group. Patients in the SR group had a higher proportion of peritoneal relapse but a lower proportion of distant lymph node metastasis. Additionally, patients in the SR group were less likely to receive surgery treatment and post-recurrence chemotherapy.

In colorectal cancer, studies have suggested that intensive follow-up leads to improved OS compared with less intensive or no follow-up (33, 34). Meanwhile, as a good surrogate of intensive follow-up, accumulating studies have also found that detection of AR helps achieve a superior survival in colorectal patients undergoing curative resection (35). While in gastric cancer, there were only 2 directly comparative studies with inconsistent outcomes (36, 37), we therefore meta-analyzed studies which evaluated the survival of patients according to the presence of cancer-related symptoms or not to investigate the relationship between intensive follow-up and survival outcomes in GC patients undergoing curative resection.

In our meta-analysis, we demonstrated that the detection of AR indeed helps improve OS in GC patients undergoing curative resection. Although there was a degree of heterogeneity in this outcome, our detailed subgroup analyses and sensitivity analysis demonstrated the stability and reliability of this result. Therefore, it is convinced that intensive follow-up should be recommended for patients undergoing radical gastrectomy, owing to the substantial survival benefit.

Intuitively, intensive follow-up is more likely to lead to early detection of recurrence, which have been confirmed in colorectal cancer (13). However, in the present study, despite an OS benefit, the time to detection of recurrence was not different between the two groups through our overall meta-analysis. For this, some scholars explained that the detection of AR may not be the same with early detection of recurrence, and AR is just less biologically aggressive than SR (22, 23). However, considering the high heterogeneity among these included studies, we further performed sensitivity analysis and a series of subgroup analyses. And a subgroup involving studies with a sample size of more than 200 indicated that patients in the AR group had a shorter RFS compared with these in the SR group with I^2^ = 7%, which indicated that detection of AR may help detect recurrence earlier. However, given that only 2 studies were incorporated into this subgroup, this result should be interpreted with caution, and more prospective studies with a substantial sample size are needed to further validate this issue.

When PRS was analyzed, we identified that the AR group had a better survival compared to the SR group, which remained consistent in all available studies. Certainly, this finding should be mainly attributed to more effective treatment delivered to AR patients (24). In our pooled analyses, we revealed that patients in the AR group had a higher proportion of tumor resection and a trend towards a higher rate of chemotherapy treatment in the AR group. This was demonstrated in the study by Sisic et al. (36), which showed that GC patients who received surgery had an improved PRS compared with these who did not. Besides, Diniz et al. (24) indicated a longer PRS of the patients who received chemotherapy when compared with these receiving basic support care. Predictably, as more effective therapies including chemotherapy, targeted therapy, immunotherapy and surgical resection become available, we believe that the PRS will be further prolonged in patients with early recurrent gastric cancer.

In addition, through our pooled analyses, we found that patients in the SR group were more likely to occur peritoneal recurrence, which is closely associated with deeper tumor invasion (38). Consistently, our clinicopathological analysis also confirmed that the SR group had more advanced T staging. Meanwhile, Diniz et al. (24) demonstrated that patients with peritoneal recurrence had a worse OS when compared with these with other recurrence patterns, which may partially explain why patients in the SR group had an inferior long-term survival. However, in cases with peritoneal recurrence, Fujiya et al. (25) revealed that patients in the AR group still had an obviously improved OS than these in the SR group (median OS: 35.9 vs. 24.0 months; P=0.039). Therefore, intensive follow-up still should not be ignored, especially for patients with a high risk of peritoneal recurrence (e.g., advanced T stage).

​A growing body of research focusing on different surveillance strategies has provided us with a more thorough understanding of how intensive follow-up facilitates the promotion of oncological outcomes in cancer patients (15, 17). A meta-analysis by Halle-Smith et al. (15) showed that in pancreatic ductal adenocarcinoma, imaging-based routine surveillance is more likely to detect recurrence at the asymptomatic stage than other routine surveillance methods, and that OS may be superior in patients whose recurrence is detected at the asymptomatic stage. Meanwhile, Chidambaram et al. (17) also demonstrated the crucial role of imaging-based planned surveillance post-esophagectomy in improving PRS. On the other hand, among our included studies, limited evidence from the study by Moorcraft et al. (29) that routinely performed surveillance with clinical assessment and tumor markers, along with endoscopy and CT scans as clinically suspected, failed to confirm the benefits of surveillance in early detection of recurrence and extended OS. Therefore, we infer that the survival benefit from follow-up may be largely attributable to imaging-based surveillance strategies.

The present study has some limitations that should be acknowledged. First, all of the included studies were retrospective in nature, which may increase the risk of selective bias. Thus, more prospective studies with a large sample size are expected to provide more credible evidence on this issue. Second, substantial heterogeneity across the included studies was found in the primary outcome measures. Even though the results of sensitivity analyses and most subgroup analyses remained unchanged, we failed to change the heterogeneity. Third, the median follow-up time among the included studies ranged from 22.5 to 169.8 months, or was not reported. The insufficiency of follow-up time may affect the occurrence of time-to-event outcomes, leading to survivor bias (39, 40). Fourth, due to the limited data comprising different surveillance strategies, we were unable to quantitatively analyze which types of follow-up protocols played a central role in the survival of GC patients.​

## Conclusions

This systematic review and meta-analysis offered encouraging evidence that detection of AR using intensive follow-up was associated with an appreciable improvement in overall survival. Therefore, it is convinced that intensive follow-up should be recommended to GC patients undergoing curative gastrectomy. However, taking into account the aforementioned limitations, more high-quality prospective studies are still required to verify the results of our study.

## Data availability statement

The original contributions presented in the study are included in the article/**Supplementary Material**. Further inquiries can be directed to the corresponding author.

## Author contributions

HY-P wrote the manuscript. HY-P, MH-Y and LH-C performed the data search and data analysis. HY-P, MH-Y and LH-C prepared figures. All authors contributed to the article and approved the submitted version.

## Funding

This study was funded by Chongqing Technology innovation and Application Development Special general project (cstc2019jscx-msxmX0194).

## Conflict of interest

The authors declare that the research was conducted in the absence of any commercial or financial relationships that could be construed as a potential conflict of interest.

## Publisher’s note

All claims expressed in this article are solely those of the authors and do not necessarily represent those of their affiliated organizations, or those of the publisher, the editors and the reviewers. Any product that may be evaluated in this article, or claim that may be made by its manufacturer, is not guaranteed or endorsed by the publisher.
