# Supplementary material for: Detection of asymptomatic recurrence following curative surgery improves survival in patients with gastric cancer: A systematic review and meta-analysis
Source: Front Oncol. 2022 Oct 26;12:1011683. doi: 10.3389/fonc.2022.1011683 (PMC9643694; doi:10.3389/fonc.2022.1011683)
Supplement: Supplementary file 1 [file DataSheet_1.pdf]

Table S1. Quality assessment of included studies using Newcastle-Ottawa Scale.

Cohort study	Representatives of the exposed cohort	Selection of the non-exposed cohort	Ascertainment of exposure	Was outcome of interest present at start of study	Comparability of cohorts on the basis of the design or analysis	Assessment of outcome	Was follow-up long enough for outcomes to occur	Adequate follow up	Total
Bennett,2005[22]	1	1	1	0	1	1	1	1	7
Bilici,2013[23]	1	1	1	0	1	0	1	1	6
Diniz,2022[24]	1	1	1	0	1	1	1	1	7
Fujiya,2016[25]	1	1	1	0	1	1	1	1	7
Kim,2010[26]	1	1	1	0	0	1	1	1	6
Kodera,2003[27]	1	1	1	0	1	1	1	1	7
Mikami,2007[28]	1	1	1	0	0	1	1	1	6
Moorcraft,2016[29]	1	1	1	0	0	1	1	1	6
Park,2021[30]	1	1	1	0	1	1	1	1	7
Villarreal-Garza,2011[31]	1	1	1	0	1	1	1	1	7
Zhao,2011[32]	1	1	1	0	1	1	1	1	7

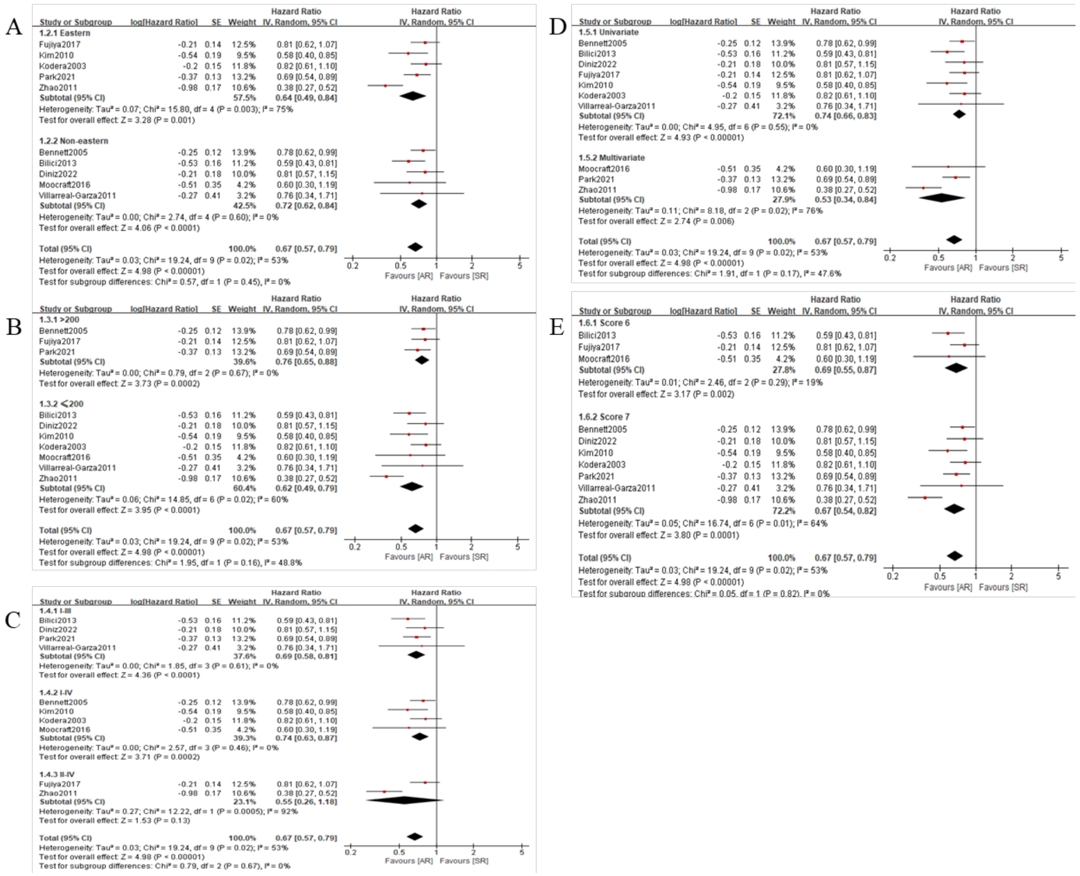

Figure S1. Forest plot of subgroup analyses assessing overall survival between the asymptomatic and symptomatic recurrence group. A: Country (Eastern vs. Non-eastern); B: Sample size (>200 vs. ≤200); C: TNM stage (I-III vs. I-IV vs. II-IV); D: Survival analysis (Univariate vs. Multivariate); E: NOS score (Score 6 vs. Score 7).

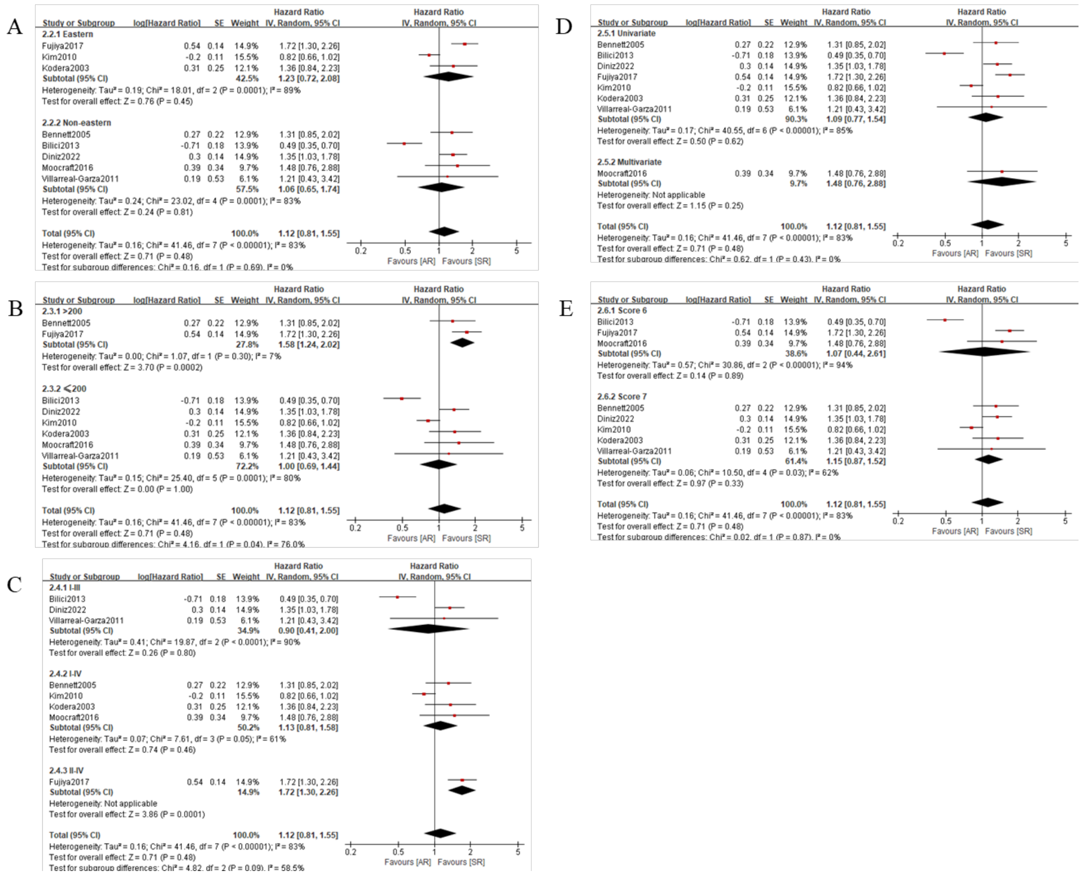

Figure S2. Forest plot of subgroup analyses assessing recurrence-free survival between the asymptomatic and symptomatic recurrence group.

A: Country (Eastern vs. Non-eastern); B: Sample size (>200 vs. ≤200); C: TNM stage (I-III vs. I-IV vs. II-IV); D: Survival analysis (Univariate vs. Multivariate); E: NOS score (Score 6 vs. Score 7).

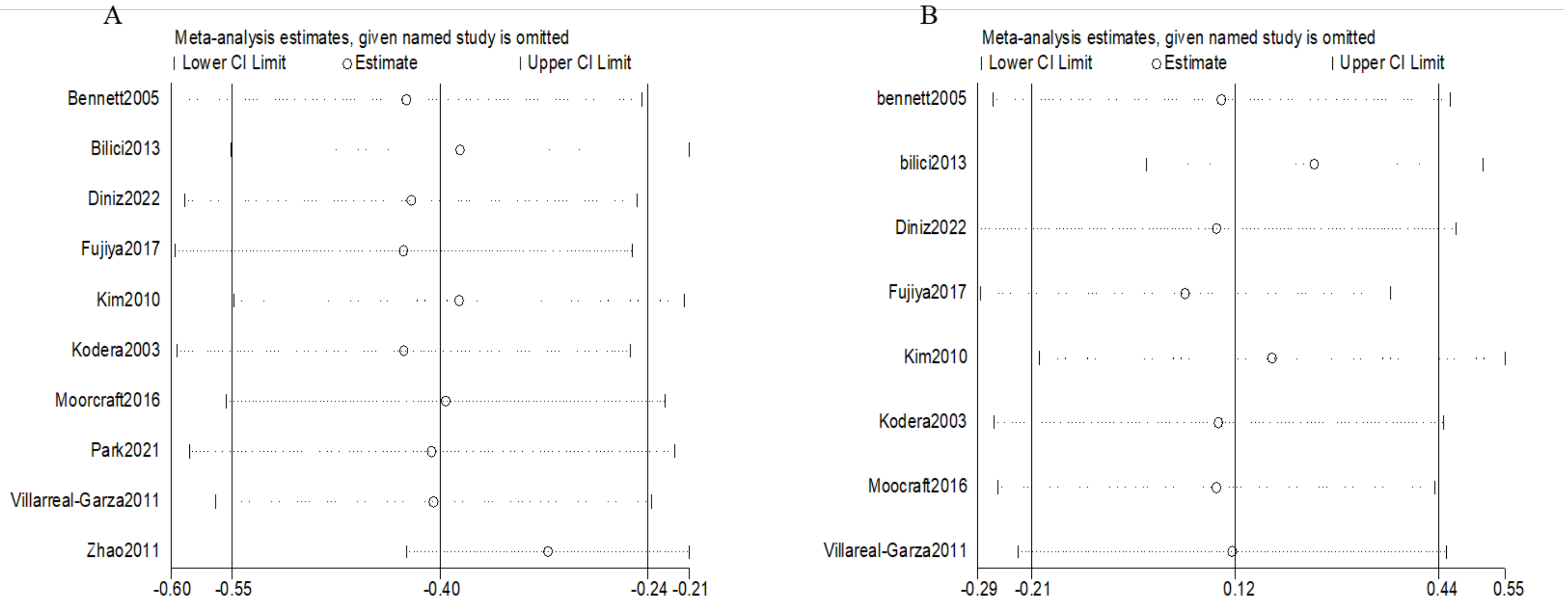

Figure S3. Sensitivity analysis of overall survival and recurrence-free survival between the asymptomatic and symptomatic recurrence group.

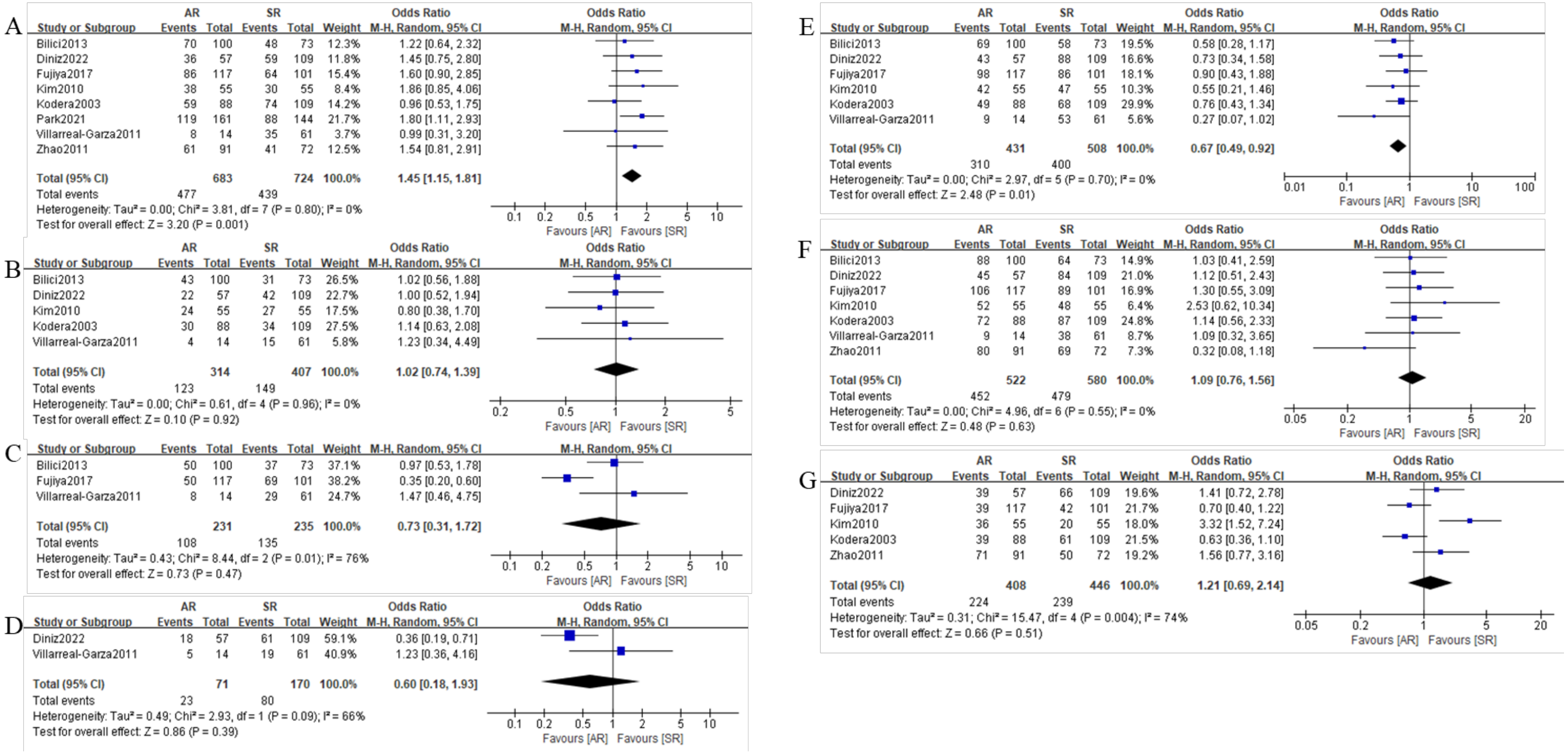

Figure S4. Forest plot assessing clinicopathological features between the asymptomatic and symptomatic recurrence group. A: sex (male); B: tumor location (lower third); C: histology differentiation (poor differentiation); D: Lauren type (intestinal type); E: T stage (III/IV); F: N stage (N+); G: adjuvant chemotherapy (yes).

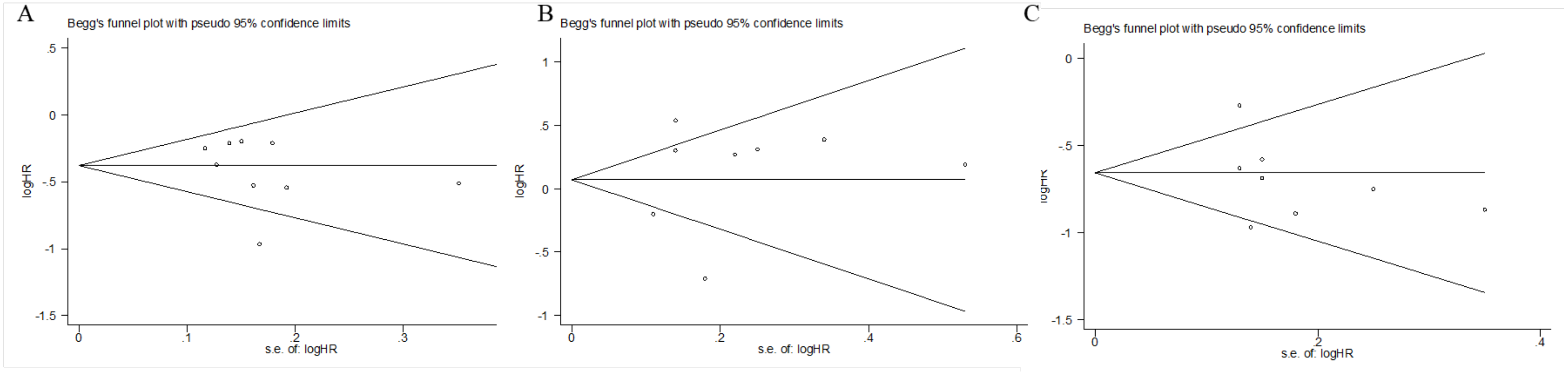

Figure S5. Funnel plots of publication bias based on A: overall survival, B: recurrence-free survival and C: post-recurrence survival did not show asymmetry. Statistical analysis suggested no evidence of publication bias with Begg’s test (P=0.371, 0.803 and 0.258, respectively).
